# Evaluating the role and effectiveness of co‐produced community‐based mental health interventions that aim to reduce suicide among adults: A systematic review

**DOI:** 10.1111/hex.13661

**Published:** 2022-11-14

**Authors:** Claire A. Hanlon, David McIlroy, Helen Poole, Jennifer Chopra, Pooja Saini

**Affiliations:** ^1^ School of Psychology, Faculty of Health Liverpool John Moores University Liverpool UK

**Keywords:** adults mental health, community‐based, co‐production, suicide, suicide prevention, systematic review

## Abstract

**Background:**

Suicide is a major public health risk requiring targeted suicide prevention interventions. The principles of co‐production are compatible with tailoring suicide prevention interventions to meet an individual's needs.

**Aims:**

This review aimed to evaluate the role and effectiveness of co‐produced community‐based suicide prevention interventions among adults.

**Methods:**

Four electronic databases (PsycInfo, CINAHL, MEDLINE and web of science) were systematically searched. A narrative synthesis was conducted.

**Results:**

From 590 papers identified through searches, 14 fulfilled the inclusion criteria. Most included studies elicited the views and perspectives of stakeholders in a process of co‐design/co‐creation of community‐based suicide prevention interventions.

**Conclusion:**

Stakeholder involvement in the creation of community‐based suicide prevention interventions may improve engagement and give voice to those experiencing suicidal crisis. However, there is limited evaluation extending beyond the design of these interventions. Further research is needed to evaluate the long‐term outcomes of co‐produced community‐based suicide prevention interventions.

**Patient and Public Involvement:**

This paper is a systematic review and did not directly involve patients and/or the public. However, the findings incorporate the views and perspectives of stakeholders as reported within the studies included in this review, and the findings may inform the future involvement of stakeholders in the design, development and delivery of community‐based suicide prevention interventions for adults.

## 1 INTRODUCTION

Co‐production is advocated within mental health policy and has garnered increasing attention.^1^, ^2^, ^3^ This is highlighted within health care initiatives including person‐centred care,^4^ the ‘Five Year Forward View for Mental Health’ policy strategy^5^ and more recently ‘The Community Mental Health Framework for Adults and Older Adults—Support, Care and Treatment. Part 1 & 2’.^6^, ^7^ Within a co‐production framework, multiple stakeholders work in collaboration, including commissioners, service providers and service users.^8^, ^9^ Emphasis is placed upon shared decision‐making and information exchange within a mutually equitable relationship.^2^ Subsequently, equal value is placed upon contributions by service users, and service providers and professionals.^2^, ^3^

It is argued that co‐production produces meaningful knowledge within the context to which it is to be applied.^9^, ^10^ This creates services that are more contextually specific, promoting engagement and bridging the translational gap between research evidence production and real‐world implementation.^9^, ^11^ Relatedly, co‐production improves quality of care,^3^, ^12^ having considered service user needs and priorities during the co‐production process^1^, ^13^ leading to cost‐efficient and cost‐effective services.^14^

Despite the highlighted benefits of co‐production, several limitations have been identified. There remains a lack of consensus in how co‐production is defined, leading to interchangeable language used to describe co‐production processes.^2^, ^13^, ^15^, ^16^ For example, undefined collaborative roles have led to a plethora of collaborative working activities marketed under a co‐production umbrella including co‐creation and co‐design.^13^, ^17^, ^18^ This ‘one size fits all’ approach is attributed to different interpretations in how co‐production is operationalized within policy, knowledge creation and subsequently implemented in practice within service delivery.^2^, ^19^, ^20^ There is a paucity of evaluation considering the extent to which co‐productive approaches cultivate meaningful outcomes^20^, ^21^, ^22^ and whether positive outcomes associated with co‐production are sustained over time.^23^ Further, reluctance to relinquish professional roles and responsibilities, such as those held by researchers or practitioners, may lead to a power imbalance that could threaten the integrity of the mutually equitable relationship.^9^, ^12^

Mental health services have striven to harness the innovative and transformative potential of co‐production in a quest to improve service user inclusivity in decision‐making, and service delivery and experience.^1^ Suicide is a major public health problem, accounting for over 700,000 deaths worldwide.^24^ Help‐seeking remains a significant barrier for those at risk of suicide, with fewer than one‐third of individuals seeking help for their mental health.^25^ The reasons why individuals experiencing suicidal thoughts and behaviours do not seek help from mental health services vary but include high self‐reliance, a low perceived need for treatment and stigmatizing attitudes towards suicide and/or mental health problems and seeking professional help.^26^ In recognition of such barriers, there has been a call for suicide prevention interventions to be tailored to improve reach and increase effectiveness.^27^

The principles of co‐production are congruent with tailoring suicide prevention interventions to suit the needs of individual service users and are aligned to recovery‐orientated services that emphasize individualized care and recognize the value of experiential knowledge.^6^, ^7^, ^28^ Research is emerging that supports implementation of co‐produced mental health service provision. For example, studies evaluating the impact of recovery colleges featuring co‐production have reported positive outcomes upon service‐user well‐being such as improved self‐esteem or confidence,^29^ improved employment opportunities^30^ and reduced use of mental health services.^31^ Additionally, applying co‐production to tailor delivery of mental health services such as the Improving Access to Psychological Therapies to improve reach among black and minority ethnic communities has shown increased accessibility and retention.^32^ Further, Pocobello et al.^33^ reported a 63.2% reduction in hospitalizations and a 39% decrease in psychiatric medication use or withdrawal among service users of an experimental co‐produced mental health service versus traditional mental health services. Findings such as these are encouraging; however, qualitative findings pervade this field and there remains a paucity of quantitative research assessing the impact of co‐production within mental health service provision,^34^ even less so in relation to suicide prevention. While studies focusing upon the preventative aspect of co‐produced mental health services assert that they prevent service user mental health from reaching crisis point,^34^ validated assessment of this impact is lacking.

As highlighted, co‐production does have its limitations, which need to be mitigated for the potential of co‐production in suicide prevention to be fully embraced. Key to furthering understanding of the role of co‐production within suicide prevention relies upon understanding the language used to define co‐production; evaluating how and to what extent service providers and service users contribute to the co‐produced service and how information is synthesized, and outcomes are assessed. Therefore, this review aims to evaluate the role and effectiveness of co‐produced, community‐based suicide prevention interventions for adults that aim to reduce suicide to:

1. Understand how co‐production is defined and operationalized.
2. Examine evidence for the role of co‐production in these interventions.
3. Identify and evaluate co‐production‐related outcomes associated with these interventions.
4. Identify and evaluate intervention components associated with a reduction in suicide‐related outcomes.

## 2 METHODS

The protocol for this review was registered on the University of York, Systematic Review database PROSPERO (CRD42020221564).^35^ The research questions and inclusion and exclusion criteria were generated using the patient/problem or population, intervention, comparator and outcome (PICO) framework.

### 2.1 Eligibility criteria

Studies were eligible for inclusion if they fulfilled the following criteria:

1. *Population*: Adults aged 18 years or older.
2. *Intervention*: Co‐produced community‐based mental health interventions that aim to reduce suicidal risk, thoughts and/or behaviour and/or those that include subanalyses for participants described as experiencing suicidal crisis or at risk of suicide were included. Treatment studies focusing upon clinical populations were excluded; however, co‐produced community‐based studies examining the effects of prevention interventions to reduce suicide risk (e.g., self‐harm, depression) were included if these data were reported as separate subanalyses. In addition, studies that broadly focussed upon mental health but clearly reported co‐produced outcomes and suicide prevention outcomes were included.
3. *Comparator*: It was unnecessary for included studies to have control group comparators. However, it was expected that some studies such as randomized‐controlled trials that fulfilled the inclusion criteria would compare intervention outcomes with a control group (e.g., usual care). Therefore, comparators could be no intervention or control group, or comparison with a different intervention group.
4. *Outcomes*: As the goal of suicide prevention interventions is to prevent suicide, changes in suicide risk and/or suicide‐related behaviours (e.g., suicide ideation) comprised the primary outcome. Both qualitative and quantitative studies (including cross‐sectional and longitudinal studies) that assessed changes in suicidal risk and behaviour were assessed against the eligibility criteria. Quantitative studies using both standardized and nonstandardized measures were eligible for inclusion. Intervention‐based studies measuring outcomes over a period of follow‐up were included only if suicide risk was reported (e.g., self‐reported) at baseline and at each follow‐up point and were re‐revaluated at follow‐up at least 1 week beyond baseline. Number of follow‐ups and type of suicide risk behaviour assessed were not determinants for inclusion. A narrative evaluation of service features of interest (e.g., co‐production definition and operationalization) was reported. Secondary outcomes were changes in psychological well‐being and quality of life.

Only studies published in English were included and no geographical or publication date restrictions were imposed. This was to capture the breath of co‐production‐based studies within the literature.

### 2.2 Search strategy

Four electronic databases (PsycINFO, CINAHL, MEDLINE, Web of Science) were searched. Studies published in English to the 21 March 2022 were eligible for inclusion. Filters were not applied during the search for type of study. Systematic reviews were excluded, but back searches of reference lists were checked for additional relevant studies that fulfilled the inclusion criteria.

### 2.3 Search terms

Scoping of the literature was undertaken in the development of the search terms exploring the extent of co‐production in the context of community mental health. Consequently, a broad search strategy was developed to ensure that all relevant papers were captured. The search strategy utilized relevant terms for co‐production (e.g., ‘co‐product*’, ‘co‐design*’, ‘co‐create’), suicide (e.g., ‘sucid*’) and community mental health (e.g., ‘community mental health’) (see Appendix A, e.g., search terms).

### 2.4 Study selection

**Figure 1:**
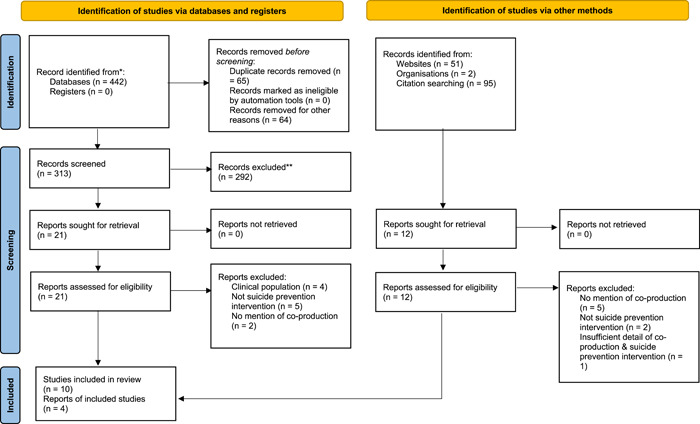
Preferred Reporting Items for Systematic Reviews and Meta‐Analyses (PRISMA) flow diagram for search outcomes and screening

The primary author removed duplicate studies from the final search and independently screened the titles and abstracts of the remaining studies against the eligibility criteria. The co‐authors also independently screened titles and abstracts according to the inclusion and exclusion criteria. Full‐text studies meeting the eligibility criteria were retrieved and reviewed for inclusion by the primary author. Two co‐authors reviewed all full‐text papers for comparison. Disagreements were resolved through discussion within the team at the title and abstract stage and by one co‐author at the full‐text screening stage. The PRISMA flowchart documents the screening process (see Figure 1). Fourteen papers were identified as eligible for inclusion.

### 2.5 Data extraction and quality assessment

Data were extracted by the primary author and transferred onto a data extraction sheet that was created and piloted before use. The following details were extracted: (1) study characteristics including study design and co‐production definition if included (Table 1) and (2) intervention characteristics including intervention type and study outcomes (Table 2).

**Table 1:** Study characteristics

References	Study aims/purpose	Design and methods (inc. measures used to assess suicide risk/behaviour)	Focus population of intervention	Age range	Community setting	Quality assessment rating
Bruce and Pearson,^44^ Country: US	To describe the aims and methodology to be used to test and evaluate the PROSPECT (Prevention of Suicide in Primary Care Elderly: Collaborative Trial) intervention, a model of depression recognition and treatment aimed at preventing and reducing suicide among older adults.	Descriptive paper, including a fictional case study, which describes a longitudinal study design planned to be used to test and evaluate the PROSPECT intervention. Proposed use of the Centers for the Epidemiologic Studies Depression (CESD) scale to screen potential participants for depression during recruitment. Eligible participants would undergo further in‐person assessment for depression and other clinical, neuropsychological and social variables. Telephone follow‐ups at 4 and 8 months and bi‐annual administration of the full research assessment battery are proposed. It is unclear what measures would determine depression‐ and suicide‐related risk/behaviours beyond screening participants for inclusion.	Community‐dwelling elderly depressed primary care patients from 18 sites within 3 geographical areas in the US were the focus population, with collaborative working between physicians and health care specialists.	Focus population age range: 780 aged 60–74 years and 600 aged 75 years and older.	18 primary care sites located in 3 geographical areas	MMAT = 20% QuADS Q = 1
Buus et al.,^49^ Country: Australia and Denmark	To examine stakeholders' suggestions and contributions to the design, function and content in the development of an existing app called MYPLAN aimed towards individuals in or at risk of suicidal crisis.	An instrumental case study involving a qualitative study using focus groups and participatory workshops.	People in or at risk of suicide crisis. Study participants, including MYPLAN app users, relatives and clinicians, worked collaboratively with the researchers and software developers revised the app.	Reported mean age range of participants: 16–46 years.	Online—A Safety planning mobile phone app	MMAT = 80% QuADS Q = 2
Cheng et al.,^50^ Country: Hong Kong	Aimed to investigate the impacts of promoting suicide prevention using social media and to evaluate the co‐creation process involving a popular YouTuber.	Mixed methods. Qualitative analysis of the co‐creation process in the development of a YouTube suicide prevention short film. Video statistics (e.g., views) generated online, an online survey and online public comments evaluated video impact and effectiveness. Suicide risk/behaviours assessed within the online survey using two questions about suicide thoughts in the past 12 months and help‐seeking.	Social media users (e.g., YouTube).	Viewers of the YouTube short film ages ranged from 13 to 44 years. Respondents filled in an online survey—ages are reported to have ranged from 12 to below 65 years.	Online—Youtube video	MMAT = 80% QuADS Q = 1
Chopra et al.,^38^ Country: UK	Aimed to evaluate the effectiveness of James' Place Model and to conduct a social value assessment of the service to provide an understanding of the potential social, economic and environmental impact of James’ Place.	Case series study involving quantitative assessment of James' Place Model effectiveness. Suicide risk assessment conducted collaboratively between a therapist and service user with a safety plan, a CORE‐OM self‐report questionnaire, referrer evaluation of precipitating factors (e.g., relationship breakdown) and therapist assessment of various psychological, motivational and volitional factors (e.g., entrapment, perceived burdensomeness).	Adult men experiencing suicidal crisis.	Adults aged 18 years and older.	Community‐based, face to face	MMAT = 80% QuADS = 1
Ferguson et al.,^39^ Country: Australia	This study aimed to explore the perspectives and experiences of workers providing case management, support or counselling to refugee and asylum seeker clients on co‐developed personalized safety plans.	Qualitative study involving semistructured interviews with workers from nongovernment organizations providing case management, support or counselling to refugees and asylum seekers.	Refugees and asylum seeker clients.	Age not given	Unclear	MMAT = 100% QuADS = 1
Hetrick et al.,^48^ Country: Australia	This study aimed to Co‐design with young people a mobile phone app‐based self‐monitoring mood tool that facilitates communication of this with a clinician.	Participatory design and studio design method were used in the development of the app, which followed human‐centred principles. This involved workshops and focus groups with young people and clinicians.	Young people experiencing depression	Young people aged 18–24 years.	Online community	MMAT = 100% QuADS = 3
Richardson et al.,^40^ Country: Ireland (both Northern & Southern Ireland)	The Young Men and Suicide Project (YMSP) aimed to develop a range of mental health initiatives to promote positive mental health among young men in Ireland and to assess the efficacy of these.	Mixed methods involving a literature review to identify best practice, online surveys with stakeholders including community‐based services, education services and prisons and focus groups service providers and men to understand what works with young men in mental health service provision. Findings informed the development and piloting of two initiatives called ‘Mind Yourself’ and ‘Work out’. Pre‐ and postmeasures of self‐esteem, depression and resilience were assessed in the Mind Yourself programme. Validated psychometric tests (e.g., six items from the General Health Questionnaire‐12 [GHQ‐12]) taken pre‐, during and postintervention in the ‘work out’ programme assess changes in mental fitness.	Young men	Northern Ireland initiative targeted adolescents (age not specified). Southern Ireland initiative targeted young men (age not specified).	School Online	MMAT = 60% QuADS = 2
Saini et al.,^41^ Country: UK	This study aimed to evaluate the effectiveness of the James' Place Mode in reducing suicidality in men using the service and to conduct a social value assessment of the service to provide an understanding of the potential social, economic and environmental impact of James' Place.	Mixed methods. Qualitative methods included semistructured interviews with men who had used the service and written responses to interview questions from a GP. Quantitative analyses of pre‐ and postoutcome data. Quantitative and qualitative findings were triangulated to understand the wider social value of James' Place. Suicide risk assessment conducted collaboratively between a therapist and service user with a safety plan, CORE‐OM self‐report questionnaire, referrer evaluation of precipitating factors (e.g., relationship breakdown) and therapist assessment of various psychological, motivational and volitional factors (e.g., entrapment, perceived burdensomeness).	Adult men experiencing suicidal crisis.	18 years and older	Community‐based, face‐to‐face delivery of a suicide prevention model	MMAT = 100% QuADS = 1
Saini et al.,^42^ Country: UK	This study aimed to evaluate the effectiveness of the James' Place Model in reducing suicidality in men over a 2‐year period and to compare the findings pre‐ and post‐COVID‐19 pandemic.	Mixed methods. Semistructured qualitive interviews with therapists. Quantitative analyses of pre‐ and post‐CORE‐OM outcome data to assess the effectiveness of the James' Place Model. Suicide risk assessment conducted collaboratively between a therapist and service user with a safety plan, CORE‐OM self‐report questionnaire, referrer evaluation of precipitating factors (e.g., relationship breakdown) and therapist assessment of various psychological, motivational and volitional factors (e.g., entrapment, perceived burdensomeness).	Adult men experiencing suicidal crisis.	18 years and older	Community‐based, face‐to‐face service temporarily moved to online delivery during the COVID‐19 pandemic	MMAT = 100% QuADS = 1
Saini et al.,^43^ Country: UK	Aimed to evaluate an innovative suicidal crisis intervention for younger men (18–30 years) versus older men (31 years and older).	Case series study involving quantitative assessment CORE‐OM scores and clinical records of psychological, motivational and volitional factors associated with participants' suicidal crisis and CORE‐OM scores. Suicide risk assessment conducted collaboratively between a therapist and service user with a safety plan, CORE‐OM self‐report questionnaire, referrer evaluation of precipitating factors (e.g., relationship breakdown) and therapist assessment of various psychological, motivational and volitional factors (e.g., entrapment, perceived burdensomeness).	Adult men experiencing suicidal crisis.	18 years and older (age range 18–66 years)	Community‐based, face‐to‐face delivery of a suicide prevention model.	MMAT = 40% QuADS = 3
Thorn et al.,^51^ Country: Australia	This study aimed to improve dissemination of and engagement with the #Chatsafe guidelines by including young people in the design and development of a social media campaign to promote safe web‐based communications about suicide. Objectives of the study were to document key elements of the co‐design process, evaluate young people's experiences of the co‐design process and capture young people's recommendations for the #Chatsafe suicide prevention campaign.	Mixed methods. Participatory co‐design approach involving 11 workshops with young people. Workshop activities included a warm‐up, co‐design activities evaluation and cooldown. At the end of each workshop, participants were invited to complete a quantitative evaluation survey including questions on demographics, perceived benefits from participation and workshop acceptability and safety. Safety protocols (e.g., wellness plan) and monitoring (e.g., workshop evaluation survey/debrief) were included.	Young people accessing the web	17–25 years	Online community	MMAT = 80% QuADS = 3
Wilcock et al.,^45^ Country: UK	Evaluation of the Offload programme, a men's rugby‐league community‐based mental health programme.	Mixed methods involving pre‐ and post‐intervention questionnaires (*n* = 699) exploring aspects related to health and well‐being (e.g., resilience, social support). Also, focus groups and case studies with men who engaged with the Offload programme. Provision was available to assess men using the Patient Health Questionnaire‐9 (PHQ9) and/or the General Anxiety Disorder scale (GAD7) if facilitators delivering the intervention were concerned about a participant's well‐being. Facilitators were also able to seek advice from a mental health clinician. These measures were not routinely given for the assessment of suicidal risk/behaviours. Men did, however, self‐report mental health conditions/diagnoses.	Community, sport‐based intervention for men experiencing mental health illness (anxiety and depression) to prevent development of complex mental illness and suicide.	Men aged 16 years or older	Community‐based	MMAT = 60% QuADS = 3
Wilcock et al.,^46^ Country: UK	This study aimed to explore stakeholder perspectives of the key design characteristics and the roles played by delivery staff in the conception and development of a community‐based men's rugby mental health programme called Offload.	Qualitative study involving one‐to‐one semistructured interviews with 18 programme designers and delivery staff.	Community, sports‐based intervention for men experiencing mental health illness (anxiety and depression) to prevent development of complex mental illness and suicide.	Intervention targets men aged 16 years or older.	Community‐based	MMAT = 100% QuADS = 2
Zealberg et al.,^47^ Country: US	To describe the development of the collaboration between emergency psychiatric services and the police.	Descriptive paper outlining development of a mobile crisis programme involving collaboration between emergency psychiatric services and the police, which includes case studies to illustrate collaboration. It is unclear how suicidal risk/behaviours were determined. However, it appears that this involved a subjective or clinical assessment (e.g., a clinical history) of the situation made by police and/or psychiatric team members responding to incidents.	Community population experiencing psychiatric crisis.	Age of the focus population not specified.	Community‐based	MMAT = 40% QuADS = 1

*Note*: MMAT refers to the Mixed Methods Appraisal Tool.^36^ QuADS Q refers to the question derived from the Quality Assessment with Diverse Studies quality assessment tools.^37^

**Table 2:** Intervention characteristics

References	Intervention details	Co‐production methodological approach	Co‐production and/or suicide‐related outcomes
Bruce and Pearson^44^	Delivery of a comprehensive treatment algorithm for depression adapted from the Agency for Health Care Police and Research (AHCPR) guidelines. Antidepressant therapy or Interpersonal Therapy (IPT), if antidepressants were unwanted by the patient, was to be recommended. A health specialist (e.g., nurse, social worker or clinical psychologist) was to ‘prompt’ physicians to facilitate timely and recommended treatment decisions by advocating for patients (e.g., obtaining and providing feedback of information on patient symptoms and treatment experiences to the physician). Education was also to be provided to patients, families and physicians on depression and suicide ideation. However, it is unclear who delivered this aspect of the intervention.	Collaboration between a health specialist (e.g., nurse, social worker or clinical psychologist) and physician to facilitate timely and targeted identification and treatment of depression among older adults. It was proposed that the health specialist would liaise with the patient, help the physician to recognize depression and make treatment recommendations within the remit of the PROSPECT intervention guidelines based upon patient information/monitoring and encourage treatment adherence among patients.	No co‐production outcomes(s) provided. Outcomes proposed to assess the effectiveness and impact of the intervention relate to depressive symptomatology (e.g., suicide ideation, hopelessness, depression and suicidal risk behaviours including substance abuse and disturbed sleep). Authors estimated that 18% of participants would experience depression at baseline. No evaluation of suicide‐related outcomes provided.
Buus et al.^49^	App‐based intervention called MYPLAN combining three preventative strategies around safety planning, help‐seeking from peers and professionals and restriction of access to lethal means. An additional feature promotes help‐seeking behaviour by including a map and directions to an emergency room nearest to the users' location.	Focus groups and participatory workshops were used to further develop the MYPLAN intervention. This involved engagement between participants, software developers and researchers in the design, evaluation and revision of MYPLAN app prototypes in response to participant feedback. Emphasis was placed upon personal experiences of using MYPLAN and evaluation of its wireframe, functionality and whether the app was culturally suited to an Australian user audience. Software developers revised and developed prototypes in response to user feedback.	Thematic analysis led to the development of 3 phases of user involvement in the development of the MYPLAN app relating to ‘suggestions of core functions’, ‘refining functions’ and ‘negotiating finish’. Increased participant engagement with researchers and software developers during the later stages of user‐involving processes as the app became increasingly revised. The revised MYPLAN app included the suicidal ideation attributes scale (SIDAS) to measure suicide ideation, a mood ratings tracker and a customizable list of personal warning signs of crisis. No evaluation of the impact of the intervention upon suicidal risk/behaviours reported.
Cheng et al.^50^	Short film designed to reduce suicidality and promote help‐seeking behaviours. The storyline of the film focused upon a suicidal university student and a taxi driver who encourages the former to seek help. Also featured is an obscured scene of a suicide method (hanging).	Co‐creation of a YouTube short film involving a popular YouTuber and researchers. To inform this process, the YouTuber engaged with literature, online material and staff and clients from a local suicide survivor service.	Thematic analyses of the co‐creation process identified three facilitating factors of ‘shared concern about youth suicide prevention’, ‘enriched knowledge of lived experience with suicide’ and ‘preserve the uniqueness of the YouTuber’, and one barrier: ‘the balance between realism and appropriateness of content’. Overall, positive perceived changes in audience suicide prevention knowledge, attitudes and behaviours reported. Mixed views received from qualitative feedback and public comments. Some respondents who had suicidal thoughts and provided qualitative feedback (*n* = 22) reported that the storyline resonated with their situation (e.g., academic and life stress; *n* = 6), one felt that the film helped to alleviate stress and another felt that it motivated them to live. Three respondents criticized the film. Public comments (*n* = 164) generally supported the film (e.g., 10.8% showed support to people in distress). Eight commentators reported past suicidal thoughts; four had attempted suicide. Two commentators with suicide intent reported abandoning their suicide plans after watching the film. One commentator displayed current suicidal thoughts and another endorsed suicide as an option.
Chopra et al.^38^	A community‐based suicide prevention intervention underpinned by three prominent suicidal theories (interpersonal theory of the suicide, collaborative assessment and management of suicidality and the integrated motivational–volitional theory of suicide). Emphasis is on the therapist and service user co‐producing the therapeutic intervention together. Brief therapeutic approaches and interventions (e.g., behavioural activation, sleep hygiene) focussed upon reducing suicidal distress and developing resilience and coping are delivered.	Co‐production of the suicide prevention intervention and safety planning with men engaged in the service and therapists delivering the James' Place Model. Co‐production with stakeholders (including academics, clinicians, commissioners, therapists and experts‐by‐experience) also informed service inception, design and delivery.	Feedback evaluations completed by 18% of men (39/212) indicated that the James' Place service was perceived as a safe and welcoming therapeutic setting and improved overall mental well‐being and coping. No formal evaluation of co‐production reported. Significant mean reduction in CORE‐OM scores for men who completed assessment and discharge questionnaires. No relationship found between the precipitating factors and levels of general distress, or between those with or without each precipitating factors.
Ferguson et al.^39^	To explore the perspectives and experiences from workers who provide case management, support or counselling to refugee and asylum seeker clients on co‐created personalized safety plans.	Co‐production discussed in the context of co‐creating safety plans. The theme from worker interviews, ‘safety planning as a co‐created, personalised activity’, highlights the workers' perspectives that safety planning should be a collaborative process and personalized to the individual.	Four themes developed: ‘Safety planning as a co‐created, personalised activity for the client’; ‘therapeutic benefits of developing a safety plan’; ‘barriers to engaging in safety planning’ and ‘strategies to enhance safety planning engagement’. Overall, these highlight the perceived facilitators, barriers and strategies to enhance safety planning as a suicide prevention intervention for refugees and asylum seekers. Benefits of co‐production reported included equitable working relationship between the client and the worker, recognition of the client's expertise and flexibility and creativity to tailor and co‐creation safety planning using alternative modes (e.g., photographs, drawings). Perceived therapeutic benefits of co‐created safety planning included increased awareness of distress triggers among clients and coping strategies, use of personalized strategies to interrupt suicidal thoughts and normalization of their suicidal experience. No formal evaluation of suicide‐related outcomes provided.
Hetrick et al.^48^	Development of a mobile phone app designed to enable monitoring of mood with feedback for users and clinicians. Users able to customize the app to suit their preferences. Features included mood monitoring (named ‘well‐being checker’) with space to record factors influencing users' mood; brief personalized interventions to support young people in the time between face‐to‐face appointments linked to the well‐being tracker such as distraction techniques to reduce stress (e.g., meditation, games and breathing techniques) and a photo album to promote positive emotion (e.g., photos, supportive messages from friends and loved ones, music playlists); lastly, a one‐touch safety feature enabling users to contact emergency services and their supporters.	Co‐design workshops with young people and two focus groups with clinicians designed to elicit information sharing and generation of concepts for the app. Young people sketched design features of the app and gained feedback from the group on their individual design. The group created a design using the best ideas from individual designs in a process called feature prioritization. This informed subsequent co‐design rounds until consolidation of the best ideas resulted in the final design. Clinicians proposed their needs and concerns of monitoring young people using an app before the co‐design workshops took place. In a second focus group with clinicians, a young person involved in the co‐design workshops presented the app wireframes and clinician feedback gained on the app design and its use in practice.	Various app features supported co‐production between the app user and clinician (e.g., the onboarding process, tailoring of trigger points within the well‐being checker). The well‐being tracker mood rating function incorporated trigger points for high distress to assess suicide risk/behaviours. No formal evaluation of the effectiveness of the app in reducing suicidal risk/behaviours was reported, but it was proposed that it could enhance help‐seeking.
Richardson et al.^40^	Northern Ireland: ‘First Instinct’ a whole community approach, aimed to encourage help‐seeking among the young men. This involved development of the ‘Mind Yourself’ brief mental health intervention; young men's advisory/reference group; training programmes for practitioners focused upon developing work with men and creation of a ‘working with men’ resource library offering off‐the‐shelf resources for practitioners. Southern Ireland: ‘Work Out’, a mental fitness app, was developed that aimed to improve help‐seeking, social connectedness and mental health literacy. Comprised of a series of brief online interventions (called ‘missions’) underpinned by cognitive behavioural therapy principles that aimed to address four areas: being practical, building confidence, taking control and being a team player.	Various components of intervention design, development and delivery involved co‐production. An advisory group of key men's health and suicide prevention representatives supported and oversaw intervention development. Local stakeholder (e.g., from community‐based services, education services, prisons and young men) views on the extent and nature of mental health/suicide prevention initiatives for young men in Ireland and the perceived facilitators and barriers of working with young men elicited through surveys and focus groups informed intervention development. Northern Ireland: Local community members delivered the Mind Yourself programme. A young men's advisory forum/reference group was set up by staff from a local organization and involved local youth leaders as ‘co‐workers’ and facilitators in its delivery. Southern Ireland intervention development involved collaborative working between developers of the Irish version of ‘work out’ and developers of the Australian version through data sharing. Focus groups involving young men provided feedback on ‘Work out’ during intervention development and testing.	Facilitators of Mind Yourself perceived the programme as effective, but some barriers were identified (e.g., literacy issues hindering questionnaire completion). Positive feedback from the young men advisory/reference group reported suggested that participants reflected positively upon their involvement (e.g., welcomed the opportunity to focus on issues affecting men in an equitable way with other stakeholders). Mind Yourself evaluation showed no significant change in pre‐ and postmeasures of self‐esteem, depression and resilience. Feedback‐suggested Work Out was perceived as acceptable and accessible. No suicide‐related outcomes reported.
Saini et al.^41^	A community‐based suicide prevention intervention underpinned by three prominent suicidal theories (interpersonal theory of the suicide, collaborative assessment and management of suicidality and integrated motivational‐volitional theory of suicide). Emphasis is on the therapist and service user co‐producing the therapeutic intervention together. Brief therapeutic approaches and interventions (e.g., behavioural activation, sleep hygiene) focussed upon reducing suicidal distress and developing resilience and coping are delivered.	Co‐production of the suicide prevention intervention and safety planning with men engaged in the service and therapists delivering the James' Place Model. Co‐production with stakeholders (including academics, clinicians, commissioners, therapists and experts‐by‐experience) also informed service inception, design and delivery.	Elements of co‐production were evident in the design and delivery of the James' Place Model. For example, men spoke of the utility of the ‘lay your cards on the table’ component for exploring factors underpinning their suicidal crisis and for exploring coping strategies, and described improved mood, motivation and family relationships. No formal evaluation of co‐production provided. Impact of the intervention on suicidal crisis evaluated using CORE‐OM scores. The initial overall mean CORE‐OM score on entry to the service was reported as 85.5 (*n* = 137) and the mean overall discharge score was reported as 38.9 (*n* = 60). The mean reduction in CORE‐OM scores was reported as 46.6. Psychological factors related to men's suicidality (e.g., impulsivity, thwarted belonginess, hopelessness) reported. No relationship between precipitating factors and general distress levels found at initial assessment, or between those with and without each precipitating factors found.
Saini et al.^42^	A community‐based suicide prevention intervention underpinned by three prominent suicidal theories (interpersonal theory of the suicide, collaborative assessment and management of suicidality and integrated motivational–volitional theory of suicide). Emphasis is on the therapist and service user co‐producing the therapeutic intervention together. Brief therapeutic approaches and interventions (e.g., behavioural activation, sleep hygiene) focussed upon reducing suicidal distress and developing resilience and coping are delivered.	Co‐production of the suicide prevention intervention and safety planning with men engaged in the service and therapists delivering the James' Place Model. Co‐production with stakeholders (including academics, clinicians, commissioners, therapists and experts‐by‐experience) also informed service inception, design and delivery.	Co‐production evidenced within therapist interviews in the management of men engaged in the service during remote delivery of the James' Place Model. Formal evaluation of co‐production was not performed. Impact of the intervention on suicidal crisis evaluated using CORE‐OM scores. Evaluation of 2‐year intervention effectiveness showed an initial overall mean CORE‐OM score on entry to the service of 86.56 (*n* = 322) and a mean overall discharge score of 35.45 (*n* = 145). The mean reduction in CORE‐OM scores was reported as 50.9. Evaluation of CORE‐OM scores suggested that the James' Place model was as effective, if not more, during COVID‐19.
Saini et al.^43^	A community‐based intervention underpinned by three prominent suicidal theories (interpersonal theory of the suicide, collaborative assessment and management of suicidality and integrated motivational–volitional theory of suicide). Emphasis is on the therapist and service user co‐producing the therapeutic intervention together. Brief therapeutic approaches and interventions (e.g., behavioural activation, sleep hygiene) focussed upon reducing suicidal distress and developing resilience and coping are delivered.	Co‐production of the suicide prevention intervention and safety planning with men engaged in the service and therapists delivering the James' Place Model. Co‐production with stakeholders (including academics, clinicians, commissioners, therapists and experts‐by‐experience) also informed service inception, design and delivery.	A clinically significant reduction in the mean CORE‐OM scores between assessment and discharge for both younger and older men engaged with the James' Place Model intervention reported. No significant difference in distress scores between younger versus older men at assessment and discharge. However, younger men showed lower levels of distress compared to older men at initial assessment and lower levels of wellness than older men at discharge. No formal evaluation of co‐production. Assessment of psychological, motivational and volitional factors reported. Younger men were less affected by entrapment, defeat not engaging in new goals and had positive attitudes towards suicide than older men at assessment. Older men at discharge were significantly more likely to have an absence of positive future thinking, less social support and entrapment than younger men.
Thorn et al.^51^	A social media campaign aiming to promote safe web‐based communication about suicide.	An iterative process of co‐design whereby learning from workshops informed the next workshop. Workshop facilitators (e.g., researchers and designers) guided design activities. Co‐design activities facilitated peer‐to‐peer mapping of young people's social media usage and communication of suicide on the web, idea generation (e.g., campaign themes and content) and testing of and feedback on the design protocol for the campaign. Three key elements comprised the co‐design process: 1. ‘Define’ involved mapping young people's social media usage, their communication about suicide and determined how young people wanted #Chatsafe guidelines to be integrated into the campaign; 2. ‘Design’ involved integrating young people's perspectives and addressing their wants and needs in the campaign development including campaign themes and delivery methods; 3. ‘User‐testing’ involved prototype testing and gaining feedback. A collaborative approach ensured participant safety (e.g., a researcher accompanied distressed participants to a private space to enact the young person's wellness plan).	Overall, co‐design workshops were perceived by participants as acceptable, beneficial and safe, although some participants reported feeling suicidal (*n* = 8) or unsure whether they felt suicidal (*n* = 6) after workshops. Findings support the feasibility of safe involvement of young people in the development of co‐designed recommendations (e.g., content and format) for a web‐based suicide prevention campaign to enhance its acceptability among young people. Positive outcomes of feelings of improved ability to communicate online about suicide and to identify others who may be at risk of suicide were reported.
Wilcock et al.^45^	Ten‐week, education‐based intervention that uses the rugby league brand to address low‐level mental health problems (e.g., low self‐esteem, depression and anxiety). Rugby‐related language is used to normalize mental health, promote intervention accessibility, acceptability, engagement and adherence. Comprised of 10 sessions (called ‘fixtures’) aimed at raising awareness of mental health problems (e.g., low self‐esteem, anxiety, depression), tackling stigma and encouraging the development of coping strategies. Sessions were comprised of two, 40‐min halves.	Coproduction is evident in the design and delivery of Offload. The design phase involved collaborative working partnerships between Rugby League Cares, State of Mind, three Rugby League Club's charitable foundations (Salford Red Devils Foundation, Warrington Wolves Foundation and Vikings Sports Foundation) and over 200 men from the targeted population who participated in interviews, focus groups and questionnaires exploring their views of mental health intervention provision. Findings from men's participation informed the intervention name, where (i.e., from rugby stadiums) and when the intervention is delivered, the language used (i.e., rugby‐centric) and the content of the intervention (e.g., type of self‐care tools to use). Foundation managers/lead, former players and coaches, officials, mental health and mindfulness specialists were involved in the delivery of Offload.	The co‐produced programme content was perceived as more relatable. Accessibility, use of nonclinical language and informal setting (i.e., rugby league stadiums) were perceived to encourage help‐seeking and to remove stigma. Additional reported benefits include increased confidence and self‐esteem, improved coping, social connectedness, increased social support, willingness to talk about mental health and reduced suicide ideation and/or attempts. Pre‐ and postintervention questionnaire findings showed positive improvement in nine outcomes reported relating to areas including coping, resilience, engagement in sport and identification of support around the men. For example, approximately three‐quarters of participants reported improved awareness of how to look after their health and well‐being, coping and better able to manage setbacks and challenges.
Wilcock et al.^46^	Ten‐week, education‐based intervention that uses the rugby league brand to address low‐level mental health problems (e.g., low self‐esteem, depression and anxiety). Rugby‐related language is used to normalize mental health, promote intervention accessibility, acceptability, engagement and adherence. Comprised of 10 sessions (called ‘fixtures’) aimed at raising awareness of mental health problems (e.g., low self‐esteem, anxiety, depression), tackling stigma and encouraging the development of coping strategies. Sessions were comprised of two, 40‐min halves.	Coproduction is evident in the design and delivery of Offload. The design phase involved collaborative working partnerships between Rugby League Cares, State of Mind, three Rugby League Club's charitable foundations (Salford Red Devils Foundation, Warrington Wolves Foundation and Vikings Sports Foundation) and over 200 men from the targeted population who participated in interviews, focus groups and questionnaires exploring their views of mental health intervention provision. Findings from men's participation informed the intervention name, where (i.e., from rugby stadiums) and when the intervention is delivered, the language used (i.e., rugby‐centric) and the content of the intervention (e.g., type of self‐care tools to use). Foundation managers/lead, former players and coaches, officials, mental health and mindfulness specialists were involved in the delivery of Offload.	Thematic analysis generated three themes reflecting the importance of co‐production in the co‐design of the intervention: ‘tacit forms of knowledge are essential to initial programme designed’; ‘stigma‐free and non‐clinical environments appeal to and engage men’ and ‘lived experience and the relatability of personal adversity’. Co‐production was perceived to improve intervention reach and engagement by using nonstigmatizing language and delivering the intervention in a nonjudgmental, nonclinical environment. Delivery of solution‐focused activities provided by men with lived experience was perceived to promote relatability and trustworthiness. Suicide‐related outcomes were not formally evaluated. Delivery of the intervention by former professional sportspeople who recalled their lived experience of mental illness/adversity was perceived to possibly promote modelling of alternative masculine behaviours that could potentially enhance mental health and help‐seeking.
Zealberg et al.^47^	An emergency psychiatry‐mobile crisis programme linking key professionals, specifically mental health professionals (e.g., Master's‐level clinicians in nursing, counselling, psychology, social work) with the police to provide mobile, crisis intervention. Clinicians supported police officers in a consultative role during police incidences involving people experiencing serious mental health illness. Clinicians would obtain a history from the individual, neighbours, family and friends, drug and alcohol use and establish trust and a therapeutic alliance with the individual. Details on three case studies are provided and intervention techniques, for example developing a rapid therapeutic alliance with a woman threatening to jump from a ledge and holding her there while police assembled a safety net below.	Collaboration between the police and clinicians allowed clinicians to liaise with the individual experiencing crisis to encourage a peaceful resolution to specific situations. This was facilitated through regular meetings with law enforcement officials, reclarification of mutual responsibilities and expectations and reviewing of critical situations. This partnership was further affirmed through debriefing of police officers following incidents, providing mental health referrals for police officers and being informal consultants.	Outcomes reported relate to three case studies and involve de‐escalation of police incidents with individuals experiencing crisis.

## 3 RESULTS

The PRISMA diagram (Figure 1) illustrates the screening process. Five hundred and ninety papers were identified by searching databases (*n* = 442) and other methods (148). After the removal of duplications and nonrelevant papers (e.g., book titles, conference submissions), 449 titles and abstracts were screened. Of these, 33 papers were retrieved for full‐text screening. Fourteen studies fulfilled the inclusion criteria.

### 3.1 Description of studies

Table 1 presents a description of the characteristics of the included studies. Studies either had a qualitative (*n* = 6), mixed methods (*n* = 6) or quantitative design (*n* = 2). Notably, some studies (*n* = 5) focused upon the delivery of suicide prevention interventions online, including via apps (e.g., mobile phone apps) (*n* = 3), YouTube (*n* = 1) or to inform safe online web‐based communications (*n* = 1). Most of the remaining studies were community‐based and delivered the intervention face‐to‐face (*n* = 9). Most studies focussed upon suicide prevention among younger to older adults aged 16 years or older (*n* = 10). One study targeted older adults aged 60 years or older (*n* = 1), another focussed upon intervention delivery for adolescents and young men (*n* = 1) and two studies did not stipulate the age of the target population (*n* = 2).

### 3.2 Methodological quality

The Mixed Methods Appraisal Tool (MMAT)^36^ and an additional question taken from the Quality Assessment with Diverse Studies (QuADS) quality assessment tool^37^ to evaluate stakeholder inclusion through co‐production, were used to assess methodological quality. All studies were independently assessed by the first author (C. A. H.) and the last author (P. S.) independently assessed the quality of 10% of the included studies. MMAT revealed a range in methodological quality assessment (see Table 1). However, most studies assessed were of high quality, with nine studies scoring 80%–100%. Studies scored low to moderate in quality in terms of co‐production inclusion, appraised using the QUADS as described. No studies were excluded from this review based on quality assessment.

### 3.3 Synthesis of findings

Findings were synthesized to produce a narrative summary describing the role of co‐production in community‐based suicide prevention interventions.

#### 3.3.1 Definition and operationalization of co‐production

Half of the studies directly refer to co‐production as a methodological approach in the design of the suicide prevention intervention.^38^, ^39^, ^41^, ^42^, ^43^, ^45^, ^46^ None of the studies provide an explicit definition of co‐production. Rather, most individual studies were found to integrate key elements of co‐production within the design and/or delivery of an intervention by involving stakeholders, representing the diverse modes in which co‐production can be applied. All studies featured stakeholders working collaboratively towards some shared goal as a function of co‐production. Most studies mention stakeholder involvement in the development and design of suicide prevention interventions (*n* = 13). In five studies^40^, ^44^, ^45^, ^46^, ^47^ stakeholders, including health professionals and those with lived experience, delivered the suicide prevention interventions. Also, in five studies, those trained to deliver the suicide prevention intervention worked collaboratively with the recipient, adapting the intervention (e.g., safety plans and talk therapy) to suit their individual needs.^38^, ^39^, ^41^, ^42^, ^43^ A diverse range of stakeholders participated in the studies. Stakeholders included health professionals, clinicians, mental health specialists, police officers,^38^, ^39^, ^40^, ^41^, ^42^, ^43^, ^44^, ^45^, ^46^, ^47^, ^48^, ^49^ community representatives including sporting representatives (e.g., ex‐rugby players) and community leaders,^38^, ^40^, ^41^, ^42^, ^43^, ^45^, ^46^ YouTubers,^50^ those who are representative of theor with lived experience/or with lived experience.^38^, ^40^, ^41^, ^42^, ^43^, ^45^, ^46^, ^48^, ^49^, ^50^, ^51^

#### 3.3.2 Facilitators of co‐production

Stakeholders mainly engaged through an iterative process to elicit their perspectives on functional aspects and/or the content of the design and development of the suicide prevention intervention (*n* = 13). This was facilitated either through focus groups/workshops^40^, ^45^, ^46^, ^48^, ^49^, ^51^ and/or one‐to‐one discussions with stakeholders including researchers, those with lived experiences and a YouTuber.^38^, ^39^, ^41^, ^42^, ^43^, ^45^, ^46^, ^50^ Seven studies^38^, ^39^, ^40^, ^41^, ^42^, ^43^, ^44^, ^47^ integrated co‐production that was discursive in nature between key partners during the delivery of the suicide prevention intervention. In Bruce and Pearson's^44^ study, a health professional was nominated to advocate for the patient and to assist physicians in the recognition of depression to allow timely intervention. In contrast, discussions around the intervention and to troubleshoot potential problems that may occur during implementation were held between local police agencies before and during intervention delivery in Zealberg et al.^47^ Conversely, co‐production informed service design and delivery of four studies focusing upon a suicide prevention intervention for men experiencing suicidal crisis.^38^, ^41^, ^42^, ^43^ Co‐production was integrated in the creation of personalized safety plans for asylum seekers and refugees.^39^

Discussions acted as a forum for rapport building, enabling improved collaboration between diverse professional disciplines and people with lived experience. For example, Zealberg et al.^47^ attribute ‘prior working discussions’ with local police agencies to redressing problems and building trust within the collaborative working relationship, a key factor in the successful implementation of their suicide prevention intervention. Studies identified that discussions among stakeholders provided an opportunity for negotiation and consensus‐seeking when addressing disagreements that may arise during intervention development or delivery.^40^, ^47^, ^48^, ^49^, ^50^ Cheng et al.^50^ report that researchers expressed concern over the inclusion of a suicide scene of hanging in the co‐creation of a suicide prevention video with a YouTuber for example. The YouTuber felt that the inclusion of this scene was imperative to maintaining the authenticity of the video's storyline. However, the YouTuber adapted the scene once the researchers explained the potential for contagion effects.

#### 3.3.3 Challenges of co‐production

The evidence highlights some challenges that may hinder the inclusion of co‐production in the design and/or implementation of suicide prevention interventions. During co‐production, both parties must be willing to engage when working collaboratively. This issue is highlighted in Ferguson et al.'s^39^ study exploring the views and perspectives of workers supporting asylum seekers and refuges in the co‐creation of safety planning. Workers perceived a lack of ‘client readiness’ to engage in safety planning (e.g., unwillingness to write a safety plan down) as a potential barrier hindering the co‐production of personalized safety planning.

A reluctance of professionals to relinquish power was evident. Hetrick et al.^48^ reported clinician resistance towards the inclusion of service users in shared decision‐making and accessing a mobile App (mApp). Similarly, Buus et al.^49^ reported that software designers included a suicidality rating scale against the wishes of stakeholders involved in the design and development of an mApp. Conversely, three studies emphasize the importance of each stakeholder maintaining the boundary of their individual area of expertise when working in partnership.^47^, ^48^, ^49^ Failure to do so could affect the safety of professionals and service users during intervention delivery^47^ and unduly burden parents/clinicians with notifications alerting them to the suicidality risk of their child/patient,^49^ particularly out of working hours.^48^ Some safeguarding concerns were highlighted. These centred around whether participation may have induced suicidal feelings and^50^, ^51^ also the implications of clinicians being alerted to client suicidality out of hours and not being able to respond to this.^48^ Similarly, Thorn et al.^51^ highlight some challenges of gaining ethical approval to undertake co‐productive methodologies in suicide prevention research, and the additional burden on resources that safety protocol development and the monitoring of stakeholder well‐being may have.

#### 3.3.4 Benefits of co‐production

Integrating co‐production within the methodological approaches provided opportunity for knowledge sharing between partners to create new knowledge that could be applied to shape aspects of the suicide prevention intervention design and/or delivery. Areas of new knowledge included the identification of gaps in existing suicide prevention approaches, the adaptation of suicide prevention interventions to better suit intervention user needs and to improve reach among the targeted population. For example, Thorn et al.^51^ used new learning generated in stakeholder workshops to inform the schedule of subsequent workshops during the design and development of a suicide prevention campaign associated with the #Chatsafe project to improve reach among the targeted population.

The consultation of stakeholders, whether they have professional or lived experience expertise, encourages consideration of suicidality and suicide‐related risk factors through a different lens. Including stakeholders with lived experience promotes reaching back to gain a deeper understanding of the issues that matter, informing the adaptation of suicide prevention interventions to suit the needs and preferences of their targeted population. This effect is reported in 12 studies.^38^, ^43^, ^45^, ^46^, ^48^, ^49^, ^50^, ^51^ Richardson et al.^40^ undertook an extensive consultative process involving an advisory group, with the views of service providers and young men considered. This revealed to the researchers the issues that men experience that may place them at risk of suicide such as ‘resistance to connection’ and ‘stigma attached to mental illness and mental health’ and ways to better engage and reach young men within community settings. This acquired new learning‐informed intervention development that engaged community partnerships and young men from the targeted population. For example, ‘train the trainer’ within the Mind Yourself intervention enabled facilitators to consider different ways of engaging the targeted population before formal delivery. Similarly, in setting up a suicide prevention service for men, diverse stakeholder views informed service inception, design and delivery of James' Place reported in Chopra et al.^38^ and Saini et al.^41^, ^42^, ^43^

New knowledge acquired through stakeholder involvement led to intervention development with content adapted to suit the targeted population. Buus et al.^49^ described how participants involved in the co‐design adapted features of their mApp‐based suicide prevention intervention. This included mood descriptors that could be customized by the user and change nonclinical language used to describe core functions of the app (e.g., ‘warning signs’ was changed to ‘well‐being checker’). This is also evident in the delivery of the James' Place Model, where co‐production is used to tailor the suicide prevention intervention to suit the individual needs of men.^38^, ^41^, ^42^, ^43^ Similarly, Ferguson et al.^39^ reported that participants in their study recognized individuals as being the expert of their own life when co‐creating and co‐developing safety plans with refugees and asylum seeker clients. Also, the rugby‐themed Offload programme^45^, ^46^ was perceived as more relatable as it was delivered by those with lived experience of mental health conditions, used nonclinical language and was implemented within an informal, nonclinical environment (i.e., Rugby stadiums). In this sense, co‐production provides voice and autonomy in decision‐making for individuals accessing a suicide prevention intervention.

### 3.4 Outcomes associated with co‐produced community‐based suicide prevention interventions

Eleven studies reported participants gaining positive and enriching experiences from their involvement in co‐production‐based methodologies irrespective of the nature of this involvement (e.g., co‐design, co‐production of the suicide prevention intervention, etc.). These included beneficial/suicide literacy,^51^ enthusiasm,^48^ therapeutic benefits including normalizing suicidal experiences and being able to identify unique triggers and coping strategies,^39^ rapport and trust building,^47^ an enriching process,^50^ sharing of experiences in focus groups/debrief,^49^ receiving psychological support within a safe and supportive therapeutic environment,^41^ improved relationships, coping and understanding of health and well‐being needs^45^ and being involved in the decision‐making process alongside the therapist during the co‐production of therapy.^38^, ^41^, ^42^

A lack of formal evaluation of outcomes associated with the suicide prevention intervention is evident. This is likely in part due to the type of studies included, the majority of which focused upon the co‐design of the intervention. Nine studies^38^, ^40^, ^41^, ^42^, ^43^, ^44^, ^45^, ^47^, ^50^ propose or report some evaluation of the intervention impact. However, only half embedded formal evaluation of outcomes pre‐ and postdelivery of the intervention.^38^, ^40^, ^41^, ^42^, ^43^, ^45^ Bruce and Pearson^44^ proposed baseline measurement of various measures in their study, including depression and social variables to allow monitoring by health professionals, and anticipated that approximately 18% of their cohort would present at baseline with suicide ideation. They go on to report that these measures would be repeated at two annual follow‐up interviews and anticipated a reduction in depressive symptomatology and suicide ideation and behaviour. Cheng et al.^50^ report that participants gained improved web‐based suicide literacy skills. Zealberg et al.^47^ provide case studies to illustrate how three lives were saved by their emergency crisis support team intervention. Richardson et al.^40^ found no significant change in self‐esteem, depression and resilience in their ‘Mind Yourself’ suicide prevention intervention. However, they report gaining a valuable understanding of barriers related to procedural aspects of intervention delivery including extending the programme duration and the need to consider literacy levels among the target population. Lastly, four studies evaluating a suicide prevention intervention specifically for men assessed pre‐ and postintervention changes using the CORE‐OM clinical assessment tool.^38^, ^41^, ^42^, ^43^

#### 3.4.1 Mechanisms of behaviour change associated with co‐production

None of the included studies explicitly identify the mechanisms of behaviour change associated with the inclusion of co‐production. Subsequently, it is impossible to determine whether any potential behaviour change related to suicide and/or mental health can be definitively attributed to the inclusion of co‐production. Nevertheless, all studies link reported outcomes to positive changes engendered by engagement in the suicide prevention intervention such as self‐monitoring of mood/well‐being,^48^ improved help‐seeking,^39^, ^42^, ^45^, ^46^, ^48^, ^49^, ^50^ rapid access ^41^, ^42^, ^44^, ^45^, ^46^, ^47^, ^48^ and improved coping strategies.^38^, ^42^, ^44^, ^45^, ^46^, ^48^, ^49^

Most studies do not specifically report on the theory underpinning suicide prevention interventions, despite a wide range of techniques being used to reduce suicidality. Four studies describe three models of suicide underpinning the suicide prevention intervention,^38^, ^41^, ^42^, ^43^ namely, the interpersonal theory of suicide,^52^ the collaborative assessment and management of suicidality^53^ and the integrated motivational–volitional theory of suicide.^54^, ^55^ However, these studies each focus upon evaluating the same suicide prevention intervention, the James' Place Model. Similarly, Hetrick et al.^48^ link the functionality of the content of their mApp to Dialectical Behavioural Therapy and Thorn et al.^51^ relate features of their #chatsafe to the resilient‐focussed Papageno effect. In addition, while not explicitly theory‐based, Buus et al.'s^49^ mApp and the safety planning intervention used by Ferguson et al.^39^ are based upon Stanley and Brown's^56^ safety planning tool.

## 4 DISCUSSION

This review has synthesized research evidence to understand how co‐production is defined and operationalized, and to examine how co‐production is implemented. In addition, the aim was to evaluate the outcomes assessed and to identify core components within community‐based suicide prevention interventions that aim to reduce suicide among adults. The study findings show that most included studies were qualitative (or were mixed methods including a qualitative element), aiming to elicit the perspectives and opinions of service users to inform the design and development of community‐based suicide prevention interventions. Few studies reported quantitative findings.

The rationale for why and how a co‐productive approach was to be implemented was mostly explained (e.g., to elicit stakeholder perspectives to inform intervention development). However, some studies omitted a clear definition of the nature of co‐production applied. This finding is consistent with the literature, where an agreed definition of co‐production is yet to be determined.^2^, ^17^, ^18^ As a result, the concept of co‐production is interpreted to mean different forms of activities, commanding different levels of involvement, responsibility and resources within shared decision‐making that are couched under the umbrella of co‐production.^16^, ^18^, ^19^ This points to a wider issue within the field of co‐production research as a lack of consensus in how to define co‐production means there is no clear metric against which to evaluate the multilevel components of co‐production. Smith et al.^13^ argue that researchers should abandon efforts to define co‐production in favour of embracing heterogeneity co‐production offers within research and instead provide a contextually specific definition suited to their research objectives. Others echo this and go further by advocating the abandonment of the pursuit for a gold standard definition of co‐production arguing that different approaches are needed to allow tailoring of the co‐productive approach to suit the context in which it is implemented.^57^ Instead, they urge researchers to be more reflective upon their application of co‐productive approaches and be more explicit in their reporting to overcome issues associated with poor operationalization of co‐production.^57^ Indeed, co‐production has been applied across different health‐related contexts including mental health.^58^ However, it is important for researchers to identify distinct measurable components of the co‐production approach used to facilitate the evaluation of any potential outcomes associated (i.e., you need to know you are evaluating to evaluate it).^2^

Involvement of stakeholders from diverse disciplines and backgrounds, and the collaborative working relationships formed were viewed as positive. Iterative discussions between stakeholders were the lynchpin to the success of this collaborative working partnership, giving voice to stakeholders in shaping the suicide prevention interventions. Equity within collaborative working partnerships in co‐production is the cornerstone of this approach.^11^, ^34^, ^59^ Yet, resistance from some researchers, developers and clinicians towards relinquishing power was evident. For example, a software developer in Thorn et al.'s^51^ study included a safety feature despite the users explicitly expressing that they wished for this feature to be omitted. This power differential is common within the co‐production literature^59^, ^60^, ^61^ and can lead to tokenistic approaches in co‐production‐based research.^59^, ^62^, ^63^ Redressing power imbalances is important for promoting a culture that empowers stakeholders, particularly service users, to share their knowledge. Failure to do so risks undermining equity within the collaborative relationship, leading to professional knowledge being prioritized over lay knowledge.^63^ However, methods to integrate key values of co‐production to avoid potential pitfalls, including power in‐balance, have been proposed (e.g., INVOLVE).^10^

Within this review, participants' preferences of intervention content challenged researchers' and clinicians' preconceived ideas of what intervention elements should be included (e.g., Hetrick et al., study).^48^ A shift away from ‘one size fits all’ approaches in suicide prevention interventions towards a tailored approach has been called for.^27^, ^64^ Co‐production offers an opportunity to work with the individual to identify and address their unmet needs in developing a tailored intervention approach to suicide prevention. Research evidence supporting the implementation of a co‐productive approach within service design and delivery of a suicide prevention intervention is emerging. This is highlighted by studies involving the James' Place Model, which aims to support men experiencing suicidal crisis and has been found to significantly reduce suicidal distress.^38^, ^41^, ^42^, ^43^ Relatedly, participants in Ferguson et al.'s^39^ study noted the value of co‐creation in formalizing personalized safety planning with their clients for the recognition of unique triggers of distress and coping strategies to mitigate this.

The focus of this review was upon co‐production within community‐based suicide prevention interventions for adults. Several papers identified within the search referred to mobile app or online suicide prevention interventions. The authors determined it to be appropriate to include these studies as technological advancement towards web‐/app‐based suicide prevention highlights a new, burgeoning community that warrants further research to understand the potential effectiveness of these types of interventions. Web‐/app‐based suicide prevention could facilitate rapid access to support for individuals experiencing suicidal crisis. However, increased accessibility may add an additional burden to those who monitor such interventions as highlighted by some included studies (e.g., Hetrick et al., study).^48^ Additionally, the very nature of web‐/app‐based suicide prevention interventions requires users to have the relevant access to technology to support their ability to access such interventions. Therefore, whilst web‐/app‐based technology provides a conduit for remote delivery of rapid suicide prevention intervention, it also may further widen health inequalities for the most vulnerable including those of low socioeconomic status and the elderly.^65^, ^66^

A key strength of this review was the broad inclusion criteria used to capture multiple modes of co‐production implementation (e.g., co‐design, co‐create, co‐production). Second, the PRISMA reporting guidelines have also been followed. Thirdly, a second reviewer has been involved during each phase of this review, thus reducing risk of bias within the results. The findings of this review should be interpreted with caution due to the small number of included papers, inclusion of only papers published in English and the homogeneity of the study populations (i.e., westernized populations). Last, while multiple modes of co‐production were included in the search criteria, the searches of databases were limited to title searches that may have led to some studies being inadvertently omitted.

### 4.1 Implications for policy and practice

The present review findings provide some evidence that co‐production can work in practice to engender positive outcomes. However, a lack of universal definition and established model for co‐production implementation may pose some problems when creating policy and practice guidance for the implementation of co‐production within suicide prevention interventions. For example, different modes and levels of stakeholder involvement in co‐production activities were evident within the included studies, but their involvement was predominantly limited to the co‐design aspect of the intervention. Stakeholder involvement generally did not extend to other stages of the research process. This finding has been reiterated in other reviews within a health‐related context,^58^ including suicide prevention.^67^ Inclusion of stakeholders within the research process before implementation of suicide prevention intervention may allow tailoring of the intervention to suit a specific service user's needs and preferences.^67^ Yet, exclusion beyond these formative stages removes the stakeholder from decision‐making processes that may be pertinent to implementation aspects of the suicide prevention intervention (e.g., delivery and intervention evaluation and impact).^67^ Co‐produced related outcomes are often context‐specific.^57^ Therefore, involvement of stakeholders within the latter stages of the research process, including the evaluation of research findings, is warranted.^67^ This could prevent tokenistic involvement of stakeholders by legitimizing the translation of their knowledge and expertise into research evidence that meets the intervention objectives, and the creation of evaluation approaches that measure meaningful impacts associated with co‐produced suicide prevention interventions.^67^

### 4.2 Implications for future research

Future research should clearly define how co‐production is implemented and formally evaluate corresponding outputs from co‐production in the delivery of suicide prevention interventions. This is important for understanding the impact on potential outcomes, if any, associated with a co‐production approach. While it is likely that there are wider impacts associated with co‐produced community‐based suicide prevention interventions, further research is needed to understand the theoretical components of co‐produced community‐based suicide prevention interventions. This would allow for the development of validated evaluation measures that can determine the intervention effects on suicide.

While some positives were reported for the inclusion of co‐production in community‐based suicide prevention interventions, particularly from the perspective of participants, there is some evidence that some professionals (e.g., clinicians) are reticent to relinquish their paternalistic roles. Future research should seek to understand the views/perspectives of those implementing co‐produced services to understand any potential barriers and facilitators to intervention delivery.

## 5 CONCLUSION

The present review found that most studies fostering a co‐productive approach within community‐based suicide prevention interventions elicit the views and perspectives of stakeholders in a process of co‐design/co‐creation. Positive evaluation attributed towards this co‐productive approach indicates some benefits in the creation of suicide prevention intervention that recognizes and values each stakeholder and redress potential power imbalances within the therapeutic relationship. This may improve engagement and give voice and control to those experiencing suicidal crisis. However, there is limited evaluation extending beyond the design aspects of the co‐productive approach to understand its effects within community‐based suicide prevention interventions.

## AUTHOR CONTRIBUTIONS

*Substantial contributions to the conceptualisation and design of the study*: Claire Hanlon, David McIlroy, Jennifer Chopra, Helen Poole and Pooja Saini.  All searches: Claire Hanlon.  *Analysis and interpretation of data*:  Claire Hanlon, David McIlroy, Jennifer Chopra, Helen Poole and Pooja Saini.  *Revising of the work critically for intellectual content and approval of the version for publication*: Claire Hanlon, David McIlroy, Jennifer Chopra, Helen Poole and Pooja Saini. All authors have read and agreed to the published version of the manuscript.

## CONFLICT OF INTEREST

The authors declare there is no conflict of interest.

## Data Availability

Data sharing is not applicable to this article as no data sets were generated or analysed during the current study.

## APPENDIX A

See Table A1.

**Table A1:** Example of search terms

	Search term	Search field
1.	co‐product*	Title search
2.	collaborat*	Title search
3.	‘collaborative approach’	Title search
4.	co‐design*	Title search
5.	co‐creat*	Title search
6.	co‐develop*	Title search
7.	co‐evaluat*	Title search
8.	‘action research’	Title search
9.	‘lived experience’	Title search
10.	‘user experience’	Title search
11.	‘user involvement’	Title search
12.	‘patient involvement’	Title search
13.	‘patient participation’	Title search
14.	‘patient engagement’	Title search
15.	‘patient cent* care’	Title search
16.	‘person cent* care’	Title search
17.	‘shared decision making’	Title search
18.	MH suicide [MESH]	Title search
19.	suicid*	Title search
20.	Suicide [keyword]	Title search
21.	MH ‘community mental health services’ [MESH]	
22.	‘community mental health services’ [keyword]	Title search
22.	1 OR 2 OR 3 OR 4 OR 5 OR 6 OR 7 OR 8 OR 9 OR 10 OR 11 OR 12 OR 13 OR 14 OR 15 OR 16 OR 17	
23.	18 OR 19 OR 20 OR 21 OR 22	
24.	23 AND 24	
